# Supplementary material for: MetaRibo-Seq measures translation in microbiomes
Source: Nat Commun. 2020 Jun 29;11:3268. doi: 10.1038/s41467-020-17081-z (PMC7324362; doi:10.1038/s41467-020-17081-z)
Supplement: Supplementary file 10 — Supplementary Data 7 [file 41467_2020_17081_MOESM10_ESM.zip › File2/Confidence_High_Taxonomy/241477_out.krona.html]

Javascript must be enabled to view this page.

members
magnitude
magnitudeUnassigned
count
unassigned
taxon
rank

241477\_out

79

79
superkingdom
2

976
phylum
79

class
79
200643


SRS011405\_contig\_number\_15112SRS015579\_contig\_number\_20502SRS019381\_contig\_number\_contig-100\_787.122878SRS043841\_contig\_number\_3660SRS044535\_contig\_number\_11851SRS051031\_contig\_number\_contig-100\_879.195738SRS140492\_contig\_number\_31916SRS144603\_contig\_number\_contig-100\_1576.100425SRS147653\_contig\_number\_1915SRS148091\_contig\_number\_13897
171549
79
order
10

171552
3
family

3
genus
838

1262925

SRS078176\_contig\_number\_13039
1
species

1
species

SRS013800\_contig\_number\_22781
1947217

1262937

SRS024009\_contig\_number\_11989
1
species

42
family
2005525


SRS050422\_contig\_number\_32367
375288
genus
42
1

species
41

SRS013521\_contig\_number\_2615SRS013687\_contig\_number\_31620SRS013940\_contig\_number\_16195SRS014287\_contig\_number\_18671SRS014613\_contig\_number\_14404SRS014683\_contig\_number\_17346SRS015431\_contig\_number\_44788SRS016381\_contig\_number\_2595SRS017247\_contig\_number\_10257SRS018888\_contig\_number\_3986SRS019445\_contig\_number\_26447SRS023914\_contig\_number\_28420SRS024435\_contig\_number\_27489SRS045244\_contig\_number\_7036SRS046068\_contig\_number\_4418SRS048164\_contig\_number\_30050SRS048981\_contig\_number\_7493SRS049402\_contig\_number\_16103SRS049995\_contig\_number\_36558SRS051610\_contig\_number\_11166SRS053356\_contig\_number\_45294SRS063489\_contig\_number\_5992SRS064557\_contig\_number\_22637SRS064757\_contig\_number\_24145SRS077589\_contig\_number\_8081SRS098655\_contig\_number\_2596SRS103987\_contig\_number\_21849SRS104036\_contig\_number\_1645SRS1041039\_contig\_number\_29869SRS1041092\_contig\_number\_9906SRS1041118\_contig\_number\_17283SRS1041134\_contig\_number\_14726SRS1041137\_contig\_number\_12302SRS1041138\_contig\_number\_1808SRS1041141\_contig\_number\_14200SRS104327\_contig\_number\_3490SRS144537\_contig\_number\_50519SRS147346\_contig\_number\_22285SRS147425\_contig\_number\_11022SRS147557\_contig\_number\_34090SRS148253\_contig\_number\_2356
46503

815
22
family


SRS015065\_contig\_number\_31113SRS015663\_contig\_number\_36465SRS019808\_contig\_number\_18860SRS020271\_contig\_number\_2011SRS064232\_contig\_number\_2415SRS075021\_contig\_number\_7217SRS078242\_contig\_number\_25575SRS1041139\_contig\_number\_5216SRS104912\_contig\_number\_14694SRS105153\_contig\_number\_24533SRS144714\_contig\_number\_27542
816
genus
22
11


SRS053398\_contig\_number\_83SRS078176\_contig\_number\_16106
46506
2
species

820

SRS1041112\_contig\_number\_6340SRS142503\_contig\_number\_46095
2
species


SRS020328\_contig\_number\_29228
1638779
1
species

821

SRS019068\_contig\_number\_85624SRS097958\_contig\_number\_11805SRS101376\_contig\_number\_8041
species
3

species
2
2292921

SRS016335\_contig\_number\_27960SRS1041091\_contig\_number\_20184

1
species

SRS045645\_contig\_number\_2645
329854

171551
family
1

1923867
1
genus


SRS142890\_contig\_number\_23262
1686296
1
species

1
family
1853231

genus
1
283168

28118

SRS045004\_contig\_number\_9093
1
species
